# Volatile Anesthetics in Ischemic Liver Injury: Enemy or Friend?

**DOI:** 10.5812/hepatmon.19880

**Published:** 2014-06-01

**Authors:** Masood Mohseni, Saeid Safari, Seyed Moayed Alavian

**Affiliations:** 1 Department of Anesthesiology, Iran University of Medical Sciences, Tehran, IR Iran; 2 Middle East Liver Diseases Center (MELD), Tehran, IR Iran; 3 Baqiyatallah Research Center for Gastroenterology and Liver Diseases, Baqiyatallah University of Medical Sciences, Tehran, IR Iran

**Keywords:** Anesthetics, Inhalation, Liver Diseases, Perioperative Care, Anesthesiology, Intraoperative Care

Ischemic liver injury occurs in a variety of clinical settings such as trauma, shock, and liver surgery (1, 2). Ischemia and subsequent reperfusion injury rapidly evolves to sinusoid endothelial cell damage, activation of Kupffer cells, inflammation, hepatocyte necrosis and finally liver dysfunction, especially in patients with preoperative liver injures (3). Liver failure is associated with a risk of poor outcome (4). Therefore, a judicious strategy for intraoperative physical and pharmacological liver protection should be implemented. A few decades ago, postoperative mortality following liver surgery or non-hepatic surgery in a patient with liver dysfunction was as high as 50% (5, 6). Substantial improvement in techniques of surgery and perioperative care has made surgery dramatically safer in these patients. Better knowledge of liver pathophysiology has made perioperative liver protection feasible (7, 8). Contribution of anesthesiologists to new horizons of safety deserves to be highlighted. High quality organ preservation during liver surgeries by hypothermia, management of intraoperative massive hemorrhage and blood transfusion, appropriate fluid management, postoperative pain control and stress reduction and recently preconditioning by using certain opioids (9-11) and volatile anesthetics (12) are examples of protective strategies implemented by anesthesiologists.

**Figure 1.**
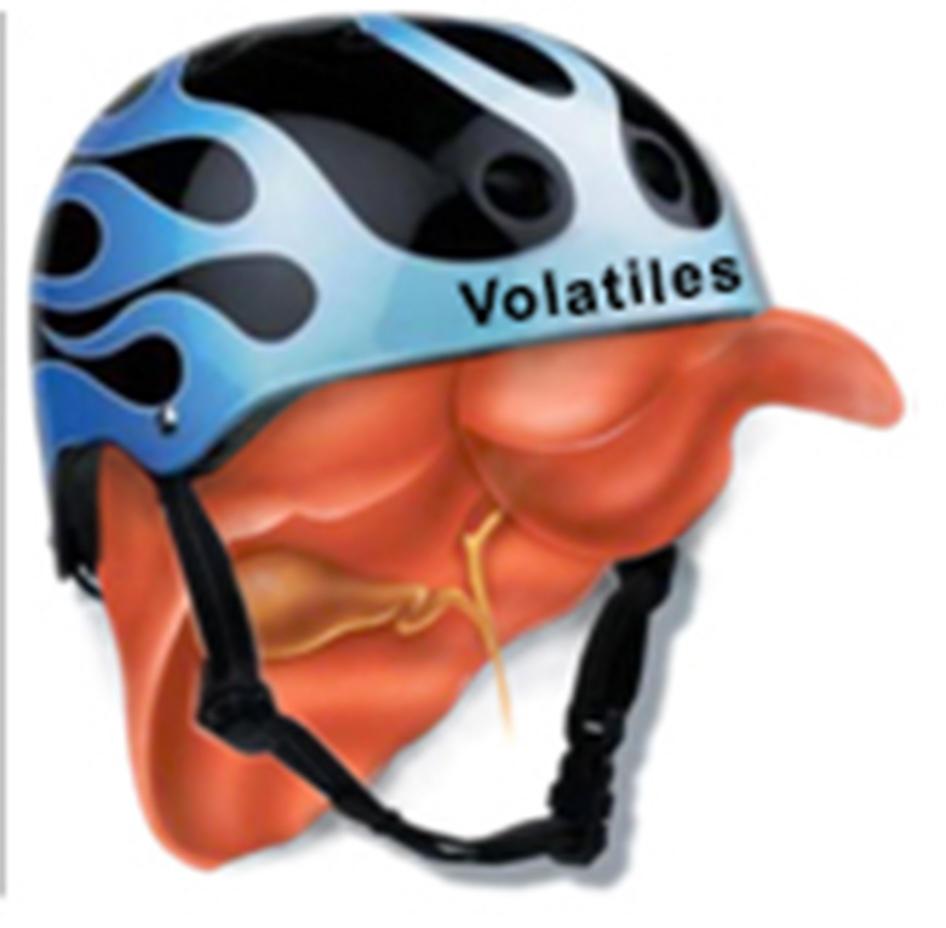
Volatile Anesthetics Should be Tolerated to Protect the Liver in Ischemic Traumas

Recent evidences suggest that an exposure to a brief period of ischemia or mild oxidative stress before a severe ischemic insult would help the organ to minimize the sequels of ischemia, a phenomenon known as preconditioning. Several modes of action have been proposed for preconditioning including biological adaptation to injury, direct protection by anti-inflammatory or anti-apoptotic mechanisms and finally organ priming by cellular activation of protection (e.g. hemoxygenases-1). A variety of preconditioning methods have been introduced to date such as hyperthermic, ischemic and pharmacological strategies, with variable degrees of efficacy (13). In liver surgery, evidences for less effectiveness of ischemic preconditioning as well as the possibility of increased intraoperative blood loss due to intermittent clamping has made ischemic preconditioning a less favorable choice compared with pharmacological preconditioning (14). Several medications have been proposed for pharmacological liver preconditioning including antioxidants, adenosine agonists, pentoxifylline, protease-inhibitors, anti-apoptotic substances, prostaglandins, matrix-metalloproteinases-inhibitors and inductors of hemoxygenases 1 (HO-1) (15). Recent laboratory and clinical studies demonstrated a promising role for volatile conditioning (12, 16) (Figure 1). A well-designed clinical trial reported that application of sevoflurane for 30 minutes before the inflow hepatic occlusion would reduce perioperative injury to hepatocytes (12). Interestingly, the protective effect of volatile anesthetics was most pronounced in patients with severe liver steatosis. Several mechanisms have been proposed for hepatoprotective effects of volatile agents including upregulation of HO-1 as an important element in the anti-oxidative system (17, 18) as well as increased production of nitric oxide (NO), demonstrated by up-regulation of inflammatory NO synthase (iNOS) (19).

Historically, it is believed that volatile anesthetics induce liver injury and impair its function. Numerous investigations have focused on hepatotoxic effects of volatile agents for half a century (5, 20-22). The story around hepatotoxic effects of volatile agents has come from findings of abnormal liver function tests and morbid outcomes following exposure to older agents such as halothane and enflurane. The incidence of autoimmune hepatitis following exposure to halothane is roughly 1:10000 (23, 24). Although controversial, enflurane has been associated with postoperative liver injury (25, 26). The pathophysiology of liver injury following exposure to halogenated anesthetics is mainly due to their metabolism to trifluoroacylated hepatic protein adducts which is a hepatotoxic compound. Concerns about decreased cardiac output and total hepatic blood flow when using volatile anesthetics have added to the problem (27). However, newer generations of volatile agents including isoflurane, desflurane and sevoflurane are usually safe because of minimal, if any, biotransformation to trifluoroacylated and even show protective effects. 

Isoflurane, an isomer of enflurane, is metabolized to a minimal degree (0.2%) and more slowly than halothane or enflurane (28). In spite of minimal biotransformation, isoflurane is accused to be the cause of a wide spectrum of perioperative liver injuries, ranging from transaminitis to fulminant hepatic failure and death (29-31). Contrary to these reports, isoflurane has been shown to protect the liver against ischemia-reperfusion injuries. Laboratory and clinical studies are still underway to clarify the exact mechanism, but isoflurane has been shown to induce an upregulation of HO-1. It seems that induction of HO-1 is related to protein kinase C and phospholipase A2 independent of nitric oxide or reactive oxygen species (32). In spite of reduced cardiac output, isoflurane also preserves hepatic blood supply, possibly due to direct vasodilatation in the hepatic vascular bed (33).

Sevoflurane does not produce acylated protein adducts and it seems that it is at least as safe as isoflurane (34). Laboratory examinations have shown the promising effect of sevoflurane on the ischemia-reperfusion injury, (35) namely during liver surgery (12).

Biotransformation of desflurane to trifluoroacylated is as low as 0.02% (36, 37). It has been shown to better preserve hepatic blood flow than earlier generations of volatile agents such as halothane and enflurane (38, 39). A recent clinical trial has suggested that the use of desflurane for maintenance of anesthesia for liver transplantation is associated with better outcome than total intravenous anesthesia (40). Collectively, most of currently available volatile anesthetics have been shown to preserve hepatic blood flow and function (41).

Liver surgery and perioperative care of patients with hepatic dysfunction is still moving toward better safety. Anesthesiologists play their role with optimal intraoperative transfusion and fluid management, pain control, stress reduction as well as judicious selection of anesthetic medications. Certain anesthetic agents such as sevoflurane and isoflurane improve hepatic perfusion and simultaneously protect the liver from ischemia-reperfusion injuries. Further research is required to confirm the hepatoprotective role of volatile agents and optimize their application in different ischemic scenarios.
